# The Beginnings of Pancreatology as a Field of Experimental and Clinical Medicine

**DOI:** 10.1155/2015/128095

**Published:** 2015-06-09

**Authors:** Piotr Ceranowicz, Jakub Cieszkowski, Zygmunt Warzecha, Beata Kuśnierz-Cabala, Artur Dembiński

**Affiliations:** ^1^Department of Physiology, Jagiellonian University Medical College, 16 Grzegorzecka Street, 31-531 Krakow, Poland; ^2^Department of Diagnostics, Chair of Clinical Biochemistry, Jagiellonian University Medical College, 15 A Kopernika Street, 31-501 Krakow, Poland

## Abstract

This review presents the history of discoveries concerning the pancreas. In antiquity and the Middle Ages knowledge about the anatomy of the pancreas was very limited and its function was completely unknown. Significant progress was first made in the seventeenth and eighteenth centuries. Johann Georg Wirsüng, the prosector of the University of Padua, discovered the main pancreatic duct, and Giovanni Santorini discovered the accessory duct. Regnier de Graaf was the first to perform pancreatic exocrine studies, and Paul Langerhans's 1869 discovery of pancreatic islets was the first step toward recognizing the pancreas as an endocrine gland. The twentieth century brought the discovery of insulin and other pancreatic hormones. To date, histochemical staining, transmission electron microscopy, and immunohistochemistry enabled the discovery of five cell types with identified hormonal products in adult human pancreatic islets. Twentieth-century pancreatic studies led to crucial advances in scientific knowledge and were recognized, among other things, with seven Nobel Prizes. The first of these went to Ivan Pavlov in 1904 for his work on the physiology of digestion. The most recent was awarded to Günter Blobel in 1999 for discovering signaling mechanisms that govern the transport and localization of proteins within pancreatic acinar cells.

## 1. Introduction

The term “pancreas” derives from Greek and consists of two words: *π*ᾶ*ν* (pan), meaning all, *κρέας* (kreas), meaning flesh. “Pancreas” thus means “all flesh” [1] and probably reflects the organ's uniform texture. It remains uncertain, however, who first discovered the pancreas and used the term “pancreas” to refer to it. The discovery is typically attributed to Herophilus of Chalcedon, who is widely considered to be the father of anatomy, and who lived at the turn of the fourth and third centuries B.C.E. [2]. Unfortunately, none of his works have been preserved, and the scope of his studies is known only from quotes that appear in the writings of later authors. Aristotle is also mentioned among the potential discoverers of the pancreas [2], but we should note that his anatomical descriptions are rather superficial, and there is no mention of the pancreas in his fundamental anatomical text, “The History of Animals” (Greek:* Περὶ τὰ ζῷα  ἱστορίαι*, Latin:* Historia Animalium*) [3].

To the best of currently available knowledge, the term “pancreas” first appears in the works of Rufus of Ephesus, a first-century Greek doctor, who was probably the first to use this term to refer to this organ [2]. We should note, however, that at the time no function was attributed to the pancreas, and Rufus of Ephesus believed that the pancreas was only an extension of the digestive system.

The Middle Ages witnessed practically no progress in pancreas-related discoveries, except for Mondino dei Luzzi's “Anatomy” [Anatomia], which went almost entirely unnoticed in the scientific world. In the course of describing two autopsies performed in 1315-16, dei Luizzi included a description that may have been referring to the pancreatic duct. Whether this was actually the case, however, remains a subject of intense controversy, and it is impossible to confirm his discovery [2].

Instead, historians give credit for discovering the pancreas to Johann Georg Wirsüng [4]. On March 2nd, 1642, at the Saint Francis Hospital in Padua, he performed an autopsy on the body of a convict who was hanged the day before, and the autopsy was witnessed by Thomas Bartholin and Moritz Hoffman [2]. On the basis of his observations, Wirsüng made a copperplate engraving showing the pancreatic duct. He then made copies and sent at least seven of them to leading anatomists of his time, including his former teacher, Professor Riolana. In the attached letters, Wirsüng noted that the pancreatic duct perforates the duodenum right next to the bile duct. He also noted that inserting a probe into the pancreatic duct from the direction of the duodenum is difficult, while insertion from the luminal surface of the duct toward the duodenum encounters no difficulties. He then reflected on the function of the pancreas and the pancreatic duct, asking whether it is a type of a vein or artery, and simultaneously answering in the negative by stating that he has never observed blood in the pancreatic duct, while the fluid present in the duct dyed the silver probe similarly to bile [2, 5].

Moritz Hoffman, who was present during the dissection performed by Wirsüng, also tried to take credit for discovering the pancreatic duct. He claimed that in 1641, a year before Wirsüng's dissection, he discovered this duct during a dissection he performed on a turkey. Hoffman also claimed that Wirsüng would not have paid attention to the duct if it had not been for their earlier conversation about the subject. The fact that Hoffman came forth with his claims five years after Wirsüng's death, and six years after the dissection during which the latter had noticed and described the pancreatic duct, makes this episode particularly controversial [2].

Ultimately, Wirsüng was credited with discovering the pancreatic duct, and it now bears his name [4]. Van Horne from Leiden was the first to use the term “Wirsüng's duct” in 1685 [2].

Likewise, the accessory pancreatic duct was discovered by the Venatian Giovanni Domenico Santorini in 1724, and it is now referred to as Santorini's duct [2, 4].

The initial steps toward discovering the role of the pancreas in digestive processes were associated with Regnier de Graaf's seventeenth-century studies [6]. He was the first to perform the groundbreaking experiment of catheterizing the pancreatic duct in a living dog. A caterer made from a quill of a wild duck made it possible to collect pancreatic juice. Unfortunately, his imperfect experimental methods limited the possibility of describing the exact function of pancreatic juice. Upon an organoleptic examination of the juice, Ragnier de Graff decided that it had a bitter aftertaste, and on this basis proposed that it participates in the process of digesting food [2].

It was the French physiologist Claude Bernard who made significant progress in specifying the function played by the pancreas in digestive processes. His research, conducted between 1846 and 1849 showed how the pancreas participates in food digestion, with particular focus on how pancreatic juice contributes to the digestion and absorption of fats [2]. Claude Bernard's experiments were both elegantly conceived and replicable, as demonstrated by de Romo and Borgstein who replicated them on the basis of his methodology in 1999 [7].

In 1869, Paul Langerhans, who studied medicine under Emile Du Bois-Reymond and was both a student and a friend of Rodolf Virchow, presented and published a dissertation entitled “Beitrage zur mikroskopischen Anatomie der Bauchspeicheldrüse” (contributions to microscopic anatomy of the pancreas) [8]. In this work he not only described the existence of acinar cells in the pancreas but also noted the presence of small, polygonal cells in the parenchyma of the pancreas; these cells had a round nucleus and no nucleolus. They stained differently than cells in their surrounding and appeared in pairs or small clusters that formed islets in the sea of acinar cells. Langerhans also noted that the islets formed by the clusters of these cells were characterized by denser innervation than the rest of the pancreas [8].

Yet the function of these islets remained unknown. In 1893, Gustave-Édouard Laguesse, a French histologist and pathologist, the first to refer to pancreatic islets as the islets of Langerhans, suggested that they might serve an endocrine function [8].

At the turn of the nineteenth and twentieth centuries, the studies of a number of scholars suggested that diabetes results from a lack of the hormone produced by the islets of Langerhans. Support for this hypothesis came from, among other things, the 1889 studies conducted by Josef von Mering and Oskar Minkowski, who showed that removing a dog's pancreas caused it to develop diabetes [9, 10]. Sometime later, Eugene Lindsay Opie found morphological changes in pancreatic islets of patients who had suffered diabetes and died of it [11, 12].

Because of the suspicion that pancreatic islets are the source of a hormone that presumably prevents diabetes, Edward Albert Sharpey-Schafer, a British physiologist and one of the founders of endocrinology, called this hypothetical hormone insulin [13]. Researchers suspected that insulin controls glucose metabolism in the body and its lack leads to increased blood sugar levels, which, in turn, leads to the appearance of sugar in urine. Attempts were made to isolate insulin from the pancreas, but because of the presence of digestive enzymes in the exocrine part of the pancreas, these attempts proved fruitless. A breakthrough came only with the work of Frederick Grant Banting. In 1921, he approached John James Rickard Macleod, a physiology professor at the University of Toronto, and proposed to conduct studies focused on insulin [14]. His suggestion was to eliminate the exocrine part of the pancreas by ligation of exocrine ducts, which could make it possible to obtain tissue which had only small amounts of digestive enzymes and which was rich in pancreatic islets. Macleod accepted Banting's proposal and, before leaving on summer break, gave him access to a lab, ten dogs, and the help of two medical students, Charles Herbert Best and E. Clark Noble [15]. Banting felt he only needed one helper, and Best and Noble decided to toss a coin. Luck favored Best. On May 16th, 1921, Banting and Best commenced a series of experiments that led to the discovery of insulin. They initially sourced insulin from dog pancreases, then from pancreases harvested from cattle embryos, and finally from ox pancreases [16]. After Macleod returned from vacation he assumed oversight over the investigations [14]. The alcohol extract that Banting obtained from pancreases lowered glucose levels in dogs that had induced diabetes, but because the extracted insulin was relatively impure, it was unsuitable for clinical studies [16], which is why an experienced biochemist, James Betram Collip, joined the team in December 1921. Collip developed a successful method that enabled the extraction of large amounts of highly purified insulin from ox pancreases, and this made it possible for a clinical study to commence in January 1922. The study was a great success and confirmed insulin's effectiveness in treating diabetes. In 1922, Banting, Best, and Collip were granted patent rights for producing insulin, but they sold them to the University of Toronto for a dollar [16]. In 1923, Frederick Grant Banting and John James Rickard Macleod were awarded the Nobel Prize in recognition of their discovery. Banting gave half of his prize money to Best, while Macleod gave half of his to Collip [14].

To the best of currently available knowledge, humans beyond the fetal stage have five types of endocrine cells in the islets of Langerhans: beta cells that produce insulin, alpha cells that produce glucagon, delta cells that produce somatostatin, PP cells that produce the pancreatic polypeptide, and ghrelin cells, responsible for the excretion of ghrelin [17]. The first study to suggest diversity among pancreatic islet cells was published by Lane in 1907 [18]. He differentiated two populations of cells, the A cells and B cells. In B cells, which are now known as beta cells, he found basophilic granules that were not present in A cells [19]. In 1957, Lacy and Davies [19] used immunohistochemical methods to show that the hormone produced by beta cells is insulin. Later researchers used electron microscopy to determine the ultrastructure of beta cells; Like performed a study of human beta cells in 1967 [20].

In 1923, Kimball and Murlin reported that the pancreas produces a hormone with hyperglycemic effects [21]. It was later named glucagon, and its molecular structure was determined in the late 1950s [22]. The exact place where glucagon was produced in the islets of Langerhans remained unknown, however, and it was only in 1962 that Hellman et al. [23] used silver staining to show that among islet cells which Lane had described as A cells (i.e., not the beta cells), there are two cell populations: A1 and A2 [23]. A2 cells were later named alpha cells [23], and immunohistochemical studies have shown that they are the glucagon production site [24, 25].

Delta cells are another type of endocrine cells found within pancreatic islets, and they were first described by Bloom in 1931 [26]. He called them D cells and showed that they stain differently than either the A or the B cells described by Lane. In 1975, several research teams used immunohistochemical methods to show that delta cells produce somatostatin [27–29].

Pancreatic polypeptide (PP) was discovered in 1968, in the course of attempts to isolate and purify chicken insulin [30]. Its presence was later confirmed in other animals and humans [31]. The main effect of pancreatic polypeptide is the inhibition of exocrine functions of the pancreas, and pancreatic enzymes in particular. PP also inhibits the contracting activity of the gall bladder and has a variety of effects on the exocrine functions of the stomach [31]. Cells that produce pancreatic polypeptide (PP cells) were initially located within the islets of Langerhans in chickens with the help of immunohistochemical methods in 1974 [32]. They were later found in humans [33] and other animals [34]. In humans, PP cells are found primarily around the perimeter of pancreatic islets, and, in small numbers, also in the parenchyma of the exocrine part of the pancreas and the epithelium of pancreatic ducts [33].

In 1999, Kojima et al. [35] found ghrelin in the gastric mucosa of humans and rats, and later studies found it in other animal species [35]. Ghrelin is produced primarily by the stomach, but it has also been found in other organs, including the intestines, pancreas, kidneys, hypothalamus, and the pituitary gland [35–37]. In adult humans ghrelin-producing cells are found in small numbers in the islets of Langerhans, where they comprise approximately 1% of the cell population [17]. A small number of ghrelin cells are also found in the epithelium of pancreatic ducts and in the parenchyma of the exocrine part of the pancreas [17]. In contrast to adulthood, during the fetal and early childhood stages of human development ghrelin cells are much more numerous and comprise approximately 10% of the population of pancreatic islet cells [17]. Ghrelin has various biological functions; stimulation of food intake [38] and excretion of the growth hormone [35] are among those that were discovered earliest. Within the pancreas, ghrelin inhibits exocrine activity [39]. Most studies also suggest that it inhibits the secretion of insulin by beta cells [17, 40–42]. Moreover, animal studies have shown that ghrelin has protective and healing effects on the pancreas. The administration of ghrelin inhibits the development of acute pancreatitis and limits damage to the pancreas [43, 44]. It also has therapeutic effects once acute pancreatitis develops and has been shown to hasten recovery [45].

## 2. Nobel Prizes

Pancreas research has led to significant developments in both pancreatology and general medicine, and since the establishment of the Nobel Prize in 1901, scientists studying the pancreas have been awarded the honor seven times [46]. Ivan Petrovich Pavlov was the first Nobel laureate to receive the prize for his work on the pancreas, among other things. He received the 1904 Nobel Prize in Physiology or Medicine, and, as the Committee wrote, it was “in recognition of his work on the physiology of digestion, through which knowledge on vital aspects of the subject has been transformed and enlarged” [47]. Pavlov's achievement consisted in discovering an automatic neurological regulation of the exocrine functions of the stomach and the pancreas. He also presented the mechanisms of the cephalic phase of pancreatic secretion, and his team discovered enterokinase and its role in the activation of trypsin [46, 48].

The second Nobel Prize awarded for pancreas research was the already mentioned 1923 prize in Physiology or Medicine that went to Frederick Grant Banting and John James Macleod for discovering insulin [14].

The third was the 1946 Chemistry prize given to John H Northrup, whose colaureates were James Batcheller Sumner and Wendell Meredith Stanley in 1946 [49]. According to the Committee's justification, Sumner received the prize “for his discovery that enzymes can be crystallized” while Northrup and Stanley received it “for their preparation of enzymes and virus proteins in a pure form” [49]. Northrup's discoveries concerning the pancreas consisted in the crystallization of trypsin and trypsinogen, and the discovery and crystallization of chymotrypsin [50].

The fourth Nobel Prize in pancreatic research was also a Chemistry prize that went to Frederick Sanger “for his work on the structure of proteins, especially that of insulin” [51].

In 1974, the Nobel Prize in Physiology or Medicine was awarded jointly to Albert Claude, Christian de Duve, and George E. Palade “for their discoveries concerning the structural and functional organization of the cell” [52]. Palade did his research on the ultrastructure of mitochondria, the endoplasmic reticulum, and ribosomes with the use of pancreatic acinar cells. He also characterized the structure of zymogen granules and examined the secretory pathway of these granules, from their formation on the rough endoplasmic reticulum, through posttranslational processing in the Golgi apparatus, to secretion out of the cell through exocytosis [53].

In 1977, Roger Guillemin, Andrew V. Schally, and Rosalyn Yalow were the corecipients of the Nobel Prize in Physiology or Medicine [54]. Guillemin and Schally received a quarter of the prize money each “for their discoveries concerning the peptide hormone production of the brain,” while the remaining half of the prize went to Rosalyn Yalow “for the development of radioimmunoassays of peptide hormones.” She used insulin in working out this method of assaying hormones [55].

The most recent and seventh Nobel Prize involving pancreas research was the 1999 Physiology or Medicine prize awarded to Günter Blobel [56], who received it “for the discovery that proteins have intrinsic signals that govern their transport and localization in the cell.” Blobel used pancreatic acinar cells in the research that made this discovery possible [57].
